# Understanding Youths’ Ability to Interpret 3D-Printed Physical Activity Data and Identify Associated Intensity Levels: Mixed-Methods Study

**DOI:** 10.2196/11253

**Published:** 2019-02-22

**Authors:** Sam Graeme Morgan Crossley, Melitta Anne McNarry, Michael Rosenberg, Zoe R Knowles, Parisa Eslambolchilar, Kelly Alexandra Mackintosh

**Affiliations:** 1 Applied Sports Science Technology and Medicine Research Centre (A-STEM) Swansea University Swansea United Kingdom; 2 School of Human Sciences The University of Western Australia Crawley Australia; 3 Physical Activity Exchange, School of Sport and Exercise Sciences Liverpool John Moores University Liverpool United Kingdom; 4 School of Computer Science and Informatics Cardiff University Cardiff United Kingdom

**Keywords:** 3D printing, education, adolescent, child, comprehension, understanding, mental recall

## Abstract

**Background:**

A significant proportion of youth in the United Kingdom fail to meet the recommended 60 minutes of moderate-to-vigorous physical activity every day. One of the major barriers encountered in achieving these physical activity recommendations is the perceived difficulty for youths to interpret physical activity intensity levels and apply them to everyday activities. Personalized physical activity feedback is an important method to educate youths about behaviors and associated outcomes. Recent advances in 3D printing have enabled novel ways of representing physical activity levels through personalized tangible feedback to enhance youths’ understanding of concepts and make data more available in the everyday physical environment rather than on screen.

**Objective:**

The purpose of this research was to elicit youths’ (children and adolescents) interpretations of two age-specific 3D models displaying physical activity and to assess their ability to appropriately align activities to the respective intensity.

**Methods:**

Twelve primary school children (9 boys; mean age 7.8 years; SD 0.4 years) and 12 secondary school adolescents (6 boys; mean age 14.1 years; SD 0.3 years) participated in individual semistructured interviews. Interview questions, in combination with two interactive tasks, focused on youths’ ability to correctly identify physical activity intensities and interpret an age-specific 3D model. Interviews were transcribed verbatim, content was analyzed, and outcomes were represented via tables and diagrammatic pen profiles.

**Results:**

Youths, irrespective of age, demonstrated a poor ability to define moderate-intensity activities. Moreover, children and adolescents demonstrated difficulty in correctly identifying light- and vigorous-intensity activities, respectively. Although youths were able to correctly interpret different components of the age-specific 3D models, children struggled to differentiate physical activity intensities represented in the models.

**Conclusions:**

These findings support the potential use of age-specific 3D models of physical activity to enhance youths’ understanding of the recommended guidelines and associated intensities.

## Introduction

Regular physical activity is considered an essential part of youths’ (children and adolescents) overall physiological health and psychosocial development [1-4], providing immediate and future health benefits [5-7]. Indeed, strong relationships exist between physical activity and health: Individuals who attain higher physical activity levels show a risk reduction of 30% for all-cause mortality, 20%-35% for cardiovascular diseases, 30%-40% for type 2 diabetes, and 20%-30% for cancer when compared to individuals who attain low activity [8]. Moreover, youths who frequently participate in physical activity demonstrate reduced symptoms of anxiety and depression, which subsequently leads to psychosocial benefits such as improved self-esteem and confidence [3]. Similar to physical activity, there is a dose-response relationship between increased sedentary behavior (activities in a sitting or reclining position such as watching television) and a greater risk of adverse health outcomes [9]. In the United Kingdom, youths aged 5-15 years have been reported to spend 7-8 hours per day in sedentary behavior, which accounts for 60%-65% of their day [10]. Given the pandemic rise of sedentary behavior in youth, public health sectors have produced and communicated physical activity recommendations to guide individuals toward achieving a minimum level of physical activity to reap health benefits [11]. The World Health Organization and UK Government both recommend that youths aged 5-17 years should engage in at least 60 minutes of moderate-to-vigorous physical activity every day [8,12]. Despite this, reports show that only 21% of boys and 16% of girls in the United Kingdom meet these physical activity recommendations [13,14].

Promotion of youth’s physical activity relies upon our understanding of the underlying factors that influence the likelihood of achieving the desired behavior. Among the most consistently reported factors are an individual’s age, sex, socioeconomic status, social and environmental support, and level of education [15-17]. However, little attention is given to individuals’ knowledge regarding the recommended levels [18-21], intensities of physical activity [22-24], and, subsequently, manners in which they achieve the international physical activity guidelines. Of concern, youths most commonly cite 2 hours per week as the recommended physical activity levels [20] and demonstrate a limited ability to interpret and classify the intensities associated with daily activities [18,25-27], thereby questioning their ability to align their own activities to the recommended levels. Furthermore, youths’ inability to define and understand the intensity of physical activity may, in part, explain the inconsistent reliability and validity of children’s self-reported physical activity levels [28-30]. Therefore, it is important to recognize youth’s lack of knowledge regarding the complexities of physical activity; content knowledge (ie, concepts) is a critical step towards youths achieving a healthy and sustainable active lifestyle that can be continued into adulthood [31]. This is particularly pertinent because adults also show a lack of knowledge of their respective physical activity targets and associated activity intensities [23]. Indeed, DiClemente et al [32] suggested that one solution to overcome youth’s lack of knowledge may be the use of personalized feedback to educate an individual about a behavior and outcome. Although there is currently a paucity of literature on youths’ current perceptions of physical activity intensity, it is evident that the development of personal feedback tools [33], which seek to enhance their understanding of the importance of physical activity and interpret the recommended guidelines, is warranted.

Digital mediums such as activity-tracking tools and mobile phone devices with assisted apps have allowed greater accessibility for users to visualize their personal physical activity data. Visualizations are known to enable users to understand their personal data and associations with physical activity levels, making them more comprehensible and actionable in terms of health-related aims [34]. However, on-screen visualizations are limited to visual stimulation and ignore the abundance of other senses, such as “touch,” that could potentially enrich personal engagement with data [34,35]. Congruent with theories built on the notion that youth are visual and tactile learners [36-38], a number of studies support the use of tangible objects to promote youth’s intellectual development [39-42]. Given that physical activity occurs in the physical world, tangible representations of physical activity that can be placed in the everyday environment have the potential to make data more available to an individual [43,44]. Indeed, Khot et al [45], investigated the use of an innovative visualization strategy involving 3D printing to create tangible physical activity data for adults, demonstrating that the visual and tactile nature of the data increased the user’s awareness and reflection of their personal physical activity behaviors. Previous evidence within the educational domains suggests that tangible interfaces can play an important role in active learning among youths by increasing engagement and reflections upon a topic [46-50]. Following these developments in understanding, recent formative research on youths has demonstrated their ability to conceptualize 3D-printed objects of physical activity, with 80% of youths expressing that the models would motivate them to engage in more physical activity [24]. Moreover, youths expressed preference for 3D models, represented through abstract and graphical designs, which led to the development of two age-specific 3D-printed model prototypes. However, before introducing the age-specific 3D models into an intervention setting [51], it is important to determine their acceptability with regard to whether youths can correctly interpret the different models in terms of the amount and intensity of daily physical activity displayed [52,53]. In the absence of such formative research, researchers risk the development of 3D models and interventions that may be inappropriate or misunderstood by the target population [54]. Indeed, previous health message interventions have been limited by a lack of formative research to guide the development and delivery of messages [55]. Based on the technology design framework developed by Druin et al [56], the present study implements the role of the “tester,” whereby youths are the testers of the new technology and their experiences can be observed and evaluated for impact by researchers.

The aims of this study were therefore to examine children’s and adolescents’ perceptions and ability to identify physical activity intensities (ie, sedentary, light, moderate, and vigorous), elicit children’s and adolescents’ interpretations of the age-specific 3D model prototypes, and use the data to consolidate the design of the age-specific 3D model prototypes to inform the development of a school-based physical activity intervention.

## Methods

### Recruitment

Participants comprised a convenience sample taken from two primary schools and two secondary schools in South Wales, United Kingdom. In total, 12 primary school children (9 boys; mean age 7.8; SD 0.4 years) and 12 secondary school adolescents (6 boys; mean age 14.1; SD 0.3 years) participated in the study. Parents and youths provided informed written consent and assent prior to participation, respectively. All procedures were approved by the University Ethics Committee and were conducted in accordance with the Declaration of Helsinki (reference no. PG/2014/40).

### Procedures

**Figure 1:**
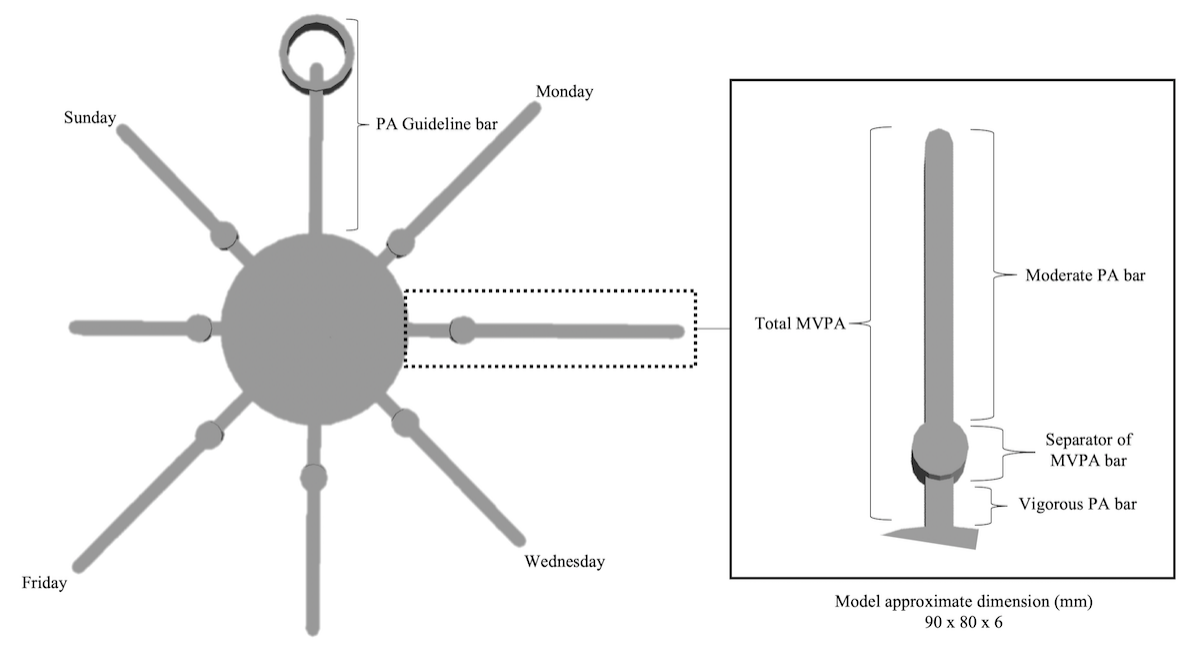
Children’s sun 3D model. PA: physical activity; MVPA: moderate-to-vigorous physical activity.

**Figure 2:**
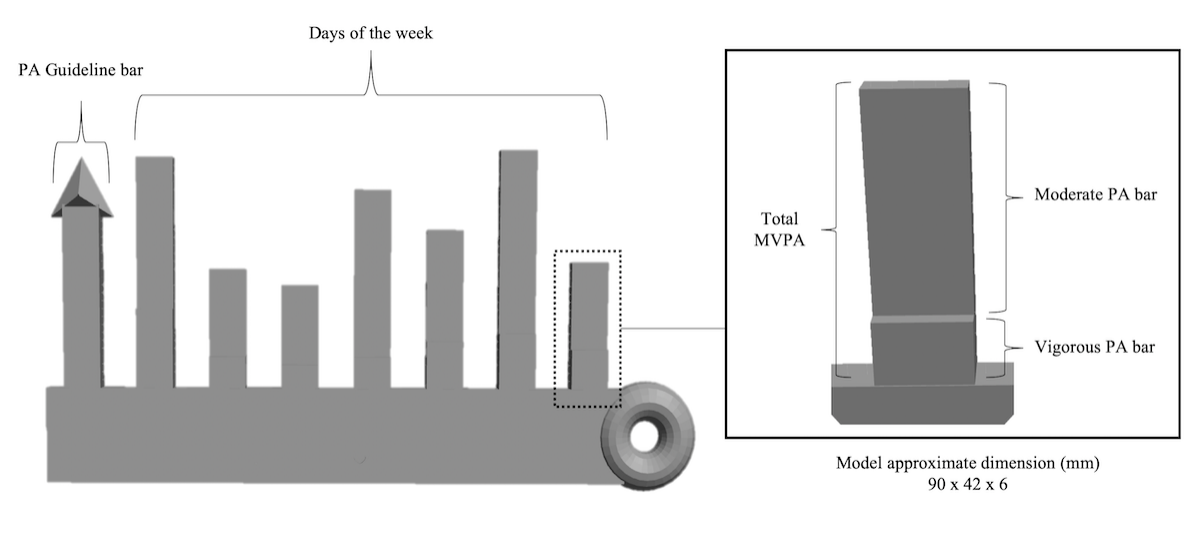
Adolescents’ bar chart 3D model. PA: physical activity; MVPA: moderate-to-vigorous physical activity.

Twenty-four semistructured individual interviews were conducted with youths by the first author, either within a familiar classroom or the school library [57]. Individual interviews are a suitable method for exploratory research seeking to generate diverse and original ideas among youths [58]. Interview questions were adjusted for tone and structure to ensure age appropriateness; all interview questions and tasks were reviewed, discussed, and revised by authors SGMC, MAM, ZRK, and KAM. The interview questions (Table 1) were informed by previous formative research [24] and addressed concepts such as youths’ knowledge of physical activity intensities and interpretations of the age-specific 3D models (Figures 1 and 2). Complementary to the interview questions, youths were asked to complete two interactive tasks: a physical activity and intensity-matching task and a 3D model recall and interpretation task. The first task was completed at the midpoint of the interview process and invited participants to match 20 different pictures of activities (eg, video gaming, walking, climbing stairs, and football) to the correct intensity (ie, sedentary, light, moderate, and vigorous; Table 2). Sedentary activities were based on the definition of Trost et al [59], whereas definitions for light, moderate, and vigorous activities were obtained from the youth compendium of physical activities [60,61]. After completion of the task, participants were asked to describe why they placed each activity within the specific intensity box.

**Table 1:** Example interview questions for children/adolescents.

Topic	Examples
Physical activity intensity	Can you tell me what you think these different levels of intensity for physical activity might be?
Physical activity intensity	What word would you use to describe the intensity of that activity (eg, climbing stairs)?
Physical activity model	What do you think the lines/bars show?
Physical activity model	Can you tell me what you think the rest of the physical activity model shows? (Prompt: how do you think this model [sun or bar chart] shows physical activity?)

**Table 2:** The 20 activities and their respective intensity levels.

Intensity	Activity
Sedentary	Eating, sitting Reading, lying down Mobile phone, sitting Computer, sitting Video games, sitting
Light (<3.0 METs^a^)	Fishing, sitting Stretching exercises Darts, wall Walking, slow
Moderate (3.0-6.0 METs^a^)	Throwing, snowball Sweeping Mowing lawn Climbing stairs
Vigorous (>6.0 METs^a^)	Climbing trees Football/soccer Tennis Hockey, field Running, hard effort Swimming laps Riding a bicycle, hard effort

^a^MET: metabolic equivalent.

The second task was completed at the end of the interview to test youths’ ability to recall and interpret the different components of the age-specific 3D models. The formatively developed 3D models were designed by youths who displayed a preference for a sun (Figure 1) and adolescents (bar chart; Figure 2) by using Play-Doh as a prototype tool for the creation [24]. Both models depict example triaxial accelerometry-derived (wGT3X-BT, ActiGraph LLC, Pensacola, FL) moderate and vigorous physical activity levels achieved for each day over a week as well as a reference bar to the physical activity guidelines of 60 minutes of moderate-to-vigorous physical activity. In detail, the moderate and vigorous physical activity level achieved for each day was calculated using Evenson’s child cut-points [62] on ActiLife, version 6.13.3 (ActiGraph LLC). Following this, the physical activity levels were inserted into the age-specific custom-developed 3D model code; loaded on OpenJSCAD, version 1.8.0; and subsequently 3D-printed using polylactide filament on the Ultimaker 2 Extended+ (Ultimaker, Geldermalsen, the Netherlands). All participants were asked to label a 2D diagram of the relevant model and verbally describe the model’s components.

Interviews lasted for a mean of 35.8 (SD 5.3) minutes and 25.1 (SD 4.9) minutes for children and adolescents, respectively. All the interviews were digitally voice recorded (Olympus DM-520 digital voice recorder, Shinjuku, Japan), video recorded (Sony Handycam HDR-PJ540, Minato, Japan), and transcribed verbatim. In total, 85 and 92 pages of raw transcription data (Arial font, size 12, double spaced) were produced for primary school children and secondary school adolescents, respectively. Unique identification codes were used to ensure anonymity of participants within all transcripts: B (boy) or G (girl), followed by participant number.

### Data Analysis

Through the process of content analysis, transcripts were deductively analyzed through contextual 3D model themes (separator of the moderate-to-vigorous physical activity bar; physical activity guideline bar; and daily, moderate, and physical activity bars) and activity intensities (sedentary, light, moderate, and vigorous intensity). Quantitatively, we analyzed whether the classification of data was accurate (ie, activities to intensities and the different 3D model components) [63]. This mixed-methods approach allowed for a greater insight into the meaning of the data [64,65] and took into account the multiple aims of the research regarding youths’ ability to identify physical activity intensities and accurately interpret the age-specific 3D models [63]. First, transcripts were thematically analyzed by the first author (SGMC) using three steps: data immersion, coding, and identifying themes [66]. The immersion of data was completed through an active way of “repeated reading” of the transcripts by searching and noting meanings and patterns within the dataset [66]. The process of coding, using a manual cut-and-paste technique, organized the data into meaningful groups that were considered pertinent to the research questions [66]. Key themes were identified by collating the relevant coded data quotes and discarding any irrelevant quotes from the analysis [66]. A frequency count of the compiled meaningful quotes was conducted to record the number of participants that noted respective points within a theme. The meaningful quotes and frequency counts were then presented diagrammatically using a pen profile approach, which is considered an appropriate method for representing diagrams of key emergent themes [67]. The last author (KAM) independently analyzed the data and discussed the outcomes with SGMC. Through the repeated process of reverse triangulation, author MAM critically cross-examined the data in reverse from the pen profiles to the transcripts until all alternative interpretations of the data were exhausted. The pen profiles were then assessed by all other authors, enabling further interpretations and adjustments before a final consensus was reached. For the activity intensity-matching task, the activities placed into certain key intensity boxes were counted (sedentary, light, moderate, and vigorous) and aligned with direct quotations (Table 3).

### Statistics

An “N−1” chi-square test was conducted using SPSS Statistics 22 (IBM Corp, Chicago, IL) to determine any significant differences between boys and girls who correctly associated activities to their respective intensity, with statistical differences accepted at *P* ≤.05 [68,69].

## Results

### Youths’ Understanding of Sedentary Behavior and Physical Activity Intensities

Youths’ understanding of physical activity intensities is presented in Tables 3 and 4 (children) and Tables 5 and 6 (adolescents), with representative verbal statements for each activity reflecting youths’ greatest intensity-level frequency count.

### Children’s Ability to Identify Sedentary Behavior and Physical Activity Intensities

Children were able to correctly align sedentary activities with the respective intensity 62% of the time, with girls demonstrating a better understanding of sedentary behavior than boys (80% vs 53%; *P*=.38). Specifically, the sedentary activities most commonly correctly identified were technology-based behaviors such as playing on a mobile phone (75%) or computer (75%) and video gaming (75%). A number of children (58%) reported that eating was a light-intensity activity: “eating’s easy cause you’re just like moving your arms and putting it [food] in your mouth” (PB06). Children were only able to correctly identify light-intensity activities 31% of the time, with girls showing a better understanding of light-intensity activities than boys (38% vs 28%; *P*=.75). A number of children (75%) indicated stretching as a moderate-intensity activity because “for some people stretching is really hard...” (PB06); one child stated, “when I do rugby you have to warm up and that’s not hard, easy or inactive” (PB07). Furthermore, fishing was identified by five children as a sedentary behavior due to the nature of the sitting position; for example, “he’s just sitting down and waiting for a fish...” (PG11). Similarly, some children struggled to define moderate-intensity activities, with only 33% of moderate activities correctly identified. Boys, as a group, fared slightly better than girls in allocating moderate-intensity activities (38% vs 25%; *P*=.66). Children perceived moderate activities such as throwing (83%), climbing stairs (75%), and sweeping (58%) as light-intensity activities. Specifically, climbing stairs was thought of as a light-intensity activity because “all you’ve got to do is lift a foot and put it on each step” (PB09), with sweeping noted as something that “you can relax while you’re doing it *”* (PB02). Vigorous activities were correctly identified 68% of the time by children (boys, 73% vs girls, 57%; *P*=.58). Vigorous-intensity activities such as riding a bicycle (92%), playing hockey (92%), playing tennis (67%), swimming laps (58%), playing football (58%), running (50%), and climbing trees (50%) were all correctly classified. Children described the nature of vigorous intensity as riding a bicycle or running, which makes one “really tired” (PB09) or “a little tired” (PB01), respectively. When referring to swimming laps, children emphasized that “my swimming teacher pushes me really hard” (PB07).

**Table 3:** Children’s ability to identify intensity of activities (n=12).

Intensity and activity item			Representative verbal statement	Frequency count according to intensity level, n					
				Sedentary		Light	Moderate	Vigorous	
**Sedentary**									
	Eating, sitting	“Eating’s easy cause you’re just like moving your arms and putting it [food] in your mouth” (PB06)			5	7^a^	0		0
	Reading, lying down	“That one cause you're just lying there” (PB03)			5^a^	3	3		1
	Mobile phone, sitting	“These [mobile phone use] are quite easy cause all you're doing is basically moving your fingers” (PB02)			9^a^	2	1		0
	Computer, sitting	“Computer you just sitting down and probably typing something with mouse and this you're just going [acts out typing]...” (PB07)			9^a^	2	1		0
	Video games, sitting	“They are like playing video games, this is inactive because you’re not actually like moving” (PB06)			9^a^	2	1		0
**Light**									
	Fishing, sitting	“He’s just sitting down and waiting for a fish but when he winds it in he’s using kind of his muscles” (PG11)			5^a^	1	4		2
	Stretching exercises	“Cause when I do rugby you have to warm up and that's not hard, easy or inactive” (PB07)			0	2	9^a^		1
	Darts, wall	“Throwing darts is pretty easy but not to hit the middle [of the dart board]” (PB02)			0	5^a^	4		3
	Walking, slow	“Walking to school’s easy, all you're doing is like moving your legs” (PB06)			1	7^a^	3		1
**Moderate**									
	Throwing, snowball	“Throwing snowballs is quite easy because you can just throw them any way you like” (PB02)			0	10^a^	2		0
	Climbing stairs	“I’ve put walking up steps because quite easy because all you've got to do is lift a foot and put it on each step” (PB09)			0	9^a^	3		0
	Sweeping	“And sweeping because you can relax while you're doing it” (PB02)			0	7^a^	5		0
	Mowing lawn	“For lawn, I've done...cause it’s not easy, and it’s not hard and it’s not inactive so it’s that one [moderate]” (PB07)			1	4	6^a^		0
**Vigorous**									
	Climbing trees	“They’re using...their tummy muscles and their arms and their legs” (PG11)			0	0	5		7^a^
	Tennis	“Then tennis cause its quite active, you move a lot cos you hit and then you have to move to hit the ball again” (PB07)			0	0	4		8^a^
	Swimming laps	“When I go swimming my teacher, go in the 3rd lane and my swimming teacher pushes me really hard” (PB07)			0	0	5		7^a^
	Hockey, field	“Hockey's hard cause some people don't really know how to play hockey...” (PB06)			0	0	1		11^a^
	Football/soccer	“...playing football's pretty hard cause...you got to get past the people who are doing skills” (PB10)			0	2	3		7^a^
	Running, hard effort	“I think running because you run a long way, you get a little tired, then you get sweaty then you can't do any more” (PB01)			0	1	5		6^a^
	Riding a bicycle, hard effort	“I did cycling because if you go really fast you might be really tired, and you might not want to do any more” (PB09)			0	0	1		11^a^

^a^Representative verbal statement frequency count.

**Table 4:** Children’s ability to correctly match activities to intensity (n=12).

Correct classification	Total (%)	Boys (%)	Girls (%)
Sedentary	62	53	80
Light	31	28	38
Moderate	33	38	25
Vigorous	68	71	57

### Adolescents’ Ability to Identify Sedentary Behavior and Physical Activity Intensities

Adolescents correctly identified sedentary-based activities 87% of the time, with boys demonstrating a better understanding than girls (90% vs 83%; *P*=.73). Sedentary technology-based activities such as playing on a mobile phone (100%) or computer (92%) and video gaming (75%) were all correctly perceived as sedentary behaviors:

They’re just on their electronics, playing games or watching something...they don’t really have to put effort into that and they're not moving around or doing anything.

SB01

Light-intensity activities were correctly identified 71% of the time, with girls displaying a better understanding than boys (75% vs 67%; *P*=.77). Light-intensity activities including walking (83%), fishing (67%), playing darts (67%), and stretching (67%) were all consistently identified as light-intensity activities. Adolescents correctly identified moderate-intensity activities only 10% of the time (girls, 13% vs boys, 8%; *P*=.07). All adolescents reported that the activity of throwing (100%) was a light-intensity activity. Other moderate activities such as mowing the lawn (75%), climbing stairs (75%), and sweeping (67%) were also classified as light-intensity activities; one adolescent described moderate activities as “everyday things like mowing the lawn” (SG09). Adolescents were only able to appropriately identify vigorous-intensity activities 46% of the time, with girls demonstrating a greater ability to recognize vigorous-intensity activities than boys (62% vs 24%; *P*=.20). Adolescents correctly categorized individual fitness activities such as cycling (75%), running (67%), and swimming (50%) as vigorous-intensity activities. In contrast, organized sport activities such as football (75%), tennis (67%), and hockey (58%) were often identified as moderate-intensity activities, although they regarded football and tennis as “...quite a physical sport” (SB03) or involving “…strengths” (SB04), respectively.

### Youths’ Understanding of the Age-Specific 3D Models

**Figure 3:**
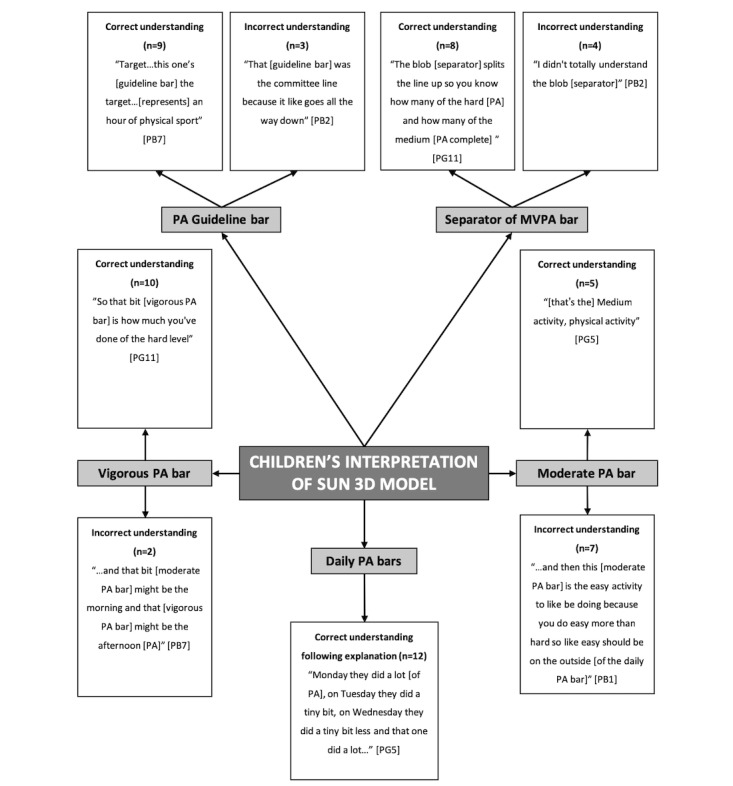
Children’s interpretation of the sun 3D model. P: primary; B: boy; G: girl; PA: physical activity; N: frequency counts; MVPA: moderate-to-vigorous physical activity.

**Figure 4:**
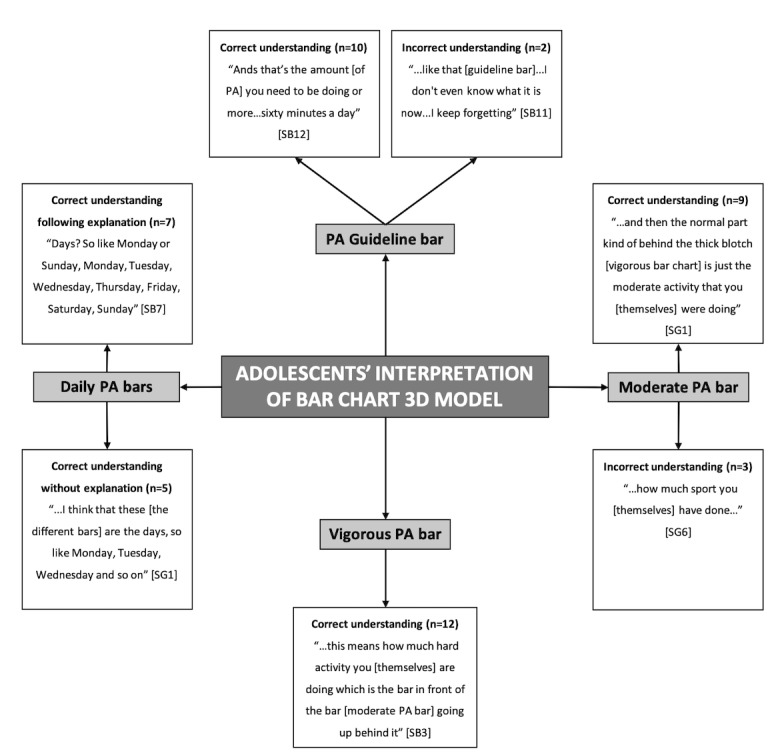
Adolescents’ interpretations of the bar chart 3D model. S: secondary; B: boy; G: girl; PA: physical activity; N: frequency counts.

Children’s and adolescents’ interpretations of the age-specific 3D models are presented in two separate pen profiles (Figures 3 and 4, respectively).

#### Children’s Understanding and Ability to Interpret the Sun 3D Model

In total, six higher-order themes were structured around the 3D model’s components: “Physical Activity Guideline Bar,” “Daily Physical Activity Bars,” “Moderate Physical Activity Bar,” “Vigorous Physical Activity Bar,” and “Separator of MVPA Bar” (Figure 3). A number of children (75%) were able to interpret the physical activity guideline bar on the 3D model as “the 60-minute time bar” (PG10). All children correctly identified that the 3D model represented a week of physical activity “Monday they did a lot [of physical activity], on Tuesday they did a tiny bit, on Wednesday they did a tiny bit less...” (PG05). The data revealed that 58% of children had some difficulty interpreting the moderate physical activity bar on the 3D model, with children describing the bar as “…the easy activity to be doing because you do easy more than hard...” (PB01). Only 42% of children were able to correctly interpret the moderate physical activity bar as “medium activity...” (PG05). Ten children (83%) correctly interpreted the vigorous physical activity bar as “how much you’ve done of the hard level [of physical activity]” (PG11), with only two children incorrectly interpreting the bar as the time at which the physical activity was undertaken: “the morning [of physical activity] and that might be the afternoon [of physical activity].” The circle separator along the sun’s rays splitting the moderate and vigorous physical activity bars was correctly interpreted by 67% of children as “the blob splits the line up, so you know how many of the hard [physical activity] and how many of the medium [physical activities]” (PG11). Only two children expressed that they did not understand the meaning of the moderate-to-vigorous separator along the ray.

#### Adolescents’ Understanding and Ability to Interpret the “Bar Chart” 3D Model

Four higher-order themes were identified around the 3D model components: “Physical Activity Guideline Bar,” “Daily Physical Activity Bars,” “Moderate Physical Activity Bar,” and “Vigorous Physical Activity Bar” (Figure 4). The physical activity guideline bar was correctly interpreted by 83% of adolescents as “that’s the amount [of physical activity] you need to be doing or more...sixty minutes a day” (SP12). Only two participants were unable to identify the meaning of the target bar. All adolescents had a good understanding of representation of the physical activity data as a week, and 42% of the adolescents were able to interpret the data without any previous explanation or guidance from the facilitator. The moderate-intensity physical activity bar was correctly reported by 75% of adolescents as “...the moderate activity that you [themselves] were doing” (SG01), with only three participants incorrectly defining it as “how much sport [they] have done” (SG6). All adolescents demonstrated a good understanding of the vigorous-intensity physical activity bar, stating “...this means how much hard activity [they] are doing...” (SB03).

**Table 5:** Adolescents’ ability to identify intensity of activities (n=12).

Intensity and activity item		Representative verbal statement	Frequency count according to intensity level, n			
			Sedentary	Light	Moderate	Vigorous
**Sedentary**						
	Eating, sitting	“Eating, maybe just a little bit of movement when you're like bringing it [the food] up to your mouth and then when you're chewing” (SB02)	10^a^	2	0	0
	Reading, lying down	“Reading a book all you're doing is just flipping a page with almost nothing movement...” (SB02)	10^a^	1	1	0
	Mobile phone, sitting	“They're just on their electronics...they don’t really have to put effort into that and they're not moving around or doing anything” (SG01)	12^a^	0	0	0
	Computer, sitting	“Yeah well obviously computer games...you’re not doing much except moving your fingers maybe” (SB02)	11^a^	1	0	0
	Video games, sitting	“Playing games...like some things that don't require that much movement” (SG10)	9^a^	2	0	0


**Light**						
	Fishing, sitting	“Fishing you’re just waiting in a boat and when a fish comes you have to reel it...” (SB02)	3	8^a^	2	0
	Stretching exercises	“It’s [stretching] not like big movement like they're not really doing much” (SB11)	2	8^a^	2	0
	Darts, wall	“Darts, all you're doing is just throwing a small dart at a small target” (SB02)	3	8^a^	1	0
	Walking, slow	“...walking to school you do need to walk obviously but it’s not very hard...” (SB02)	3^a^	1	0	0
**Moderate**						
	Throwing, snowball	“Throwing a snowball not much at all, all you have to do is just craft this little ball of precipitation and throw it at someone else” (SB02)	0	12^a^	0	0
	Climbing stairs	“Like walking up the stairs, it’s sort of easy... you can get a bit out of breath” (SB04)	0	9^a^	3	0
	Sweeping	“They're just like doing something simple, like their daily life” (SG05)	4	8^a^	0	0
	Mowing lawn	“Light is mostly just...everyday things like mowing the lawn” (SG09)	1	9^a^	2	0

**Vigorous**						
	Climbing trees	“Climbing a tree cause it does take a lot of effort to climb a tree” (SG01)	0	5	6^a^	1
	Tennis	“Just some like basic sports...people would think they're fairly easy...running, football and tennis” (SG10)	0	0	8^a^	4
	Swimming laps	“Swimming...you have to be able to do the right streamlined technique to be able to glide through the water and then...you need to be able to breathe...” (SB02)	0	2	4	6^a^
	Hockey, field	“A girl playing hockey you need to run around the pitch many times and it might get a bit tiring” (SB02)	0	0	7^a^	5
	Football/soccer	“I put quite a few in medium because like football is quite a physical sport” (SB03)	0	0	9^a^	3
	Running, hard effort	“These are probably the ones like make you push yourself” (SB11)	0	2	2	8^a^
	Riding a bicycle, hard effort	“Like cycling when you’re going up hills and stuff, it depends like how strong you are...” (SB04)	0	0	3	9^a^

^a^Representative verbal statement frequency count.

**Table 6:** Adolescents’ ability to correctly match activities to intensity (n=12).

Correct classification	Total (%)	Boys (%)	Girls (%)
Sedentary	87	90	83
Light	71	67	75
Moderate	10	8	13
Vigorous	43	24	62

## Discussion

### Principal Findings

The aims of this study were to ascertain youths’ understanding of the age-specific 3D model designs and to examine youths’ perceptions and ability to identify activities according to their respective intensity. The study findings suggest that youths demonstrate misconceptions in defining different activity intensities. However, youths’ ability to interpret the age-specific 3D models supports the use of these formatively designed tangible representations of physical activity within an intervention to aid youths’ understanding and awareness of the recommended 60 minutes of moderate-to-vigorous physical activity [24].

### Overview

To date, little research has explored how youths understand the meaning of the term *physical activity* [27,59,70,71]. It has previously been suggested that the terminology developed by adults used to describe physical activity is too complicated for youths, due to developmental and vocabulary differences [27]. As highlighted by Pearce et al [27], understanding how children express physical activity is a logical first step for improving overall knowledge and the development of innovative methods for enhancing physical activity. In the present study, the intensity-matching task revealed that adolescents have a greater ability to identify sedentary behaviors and light-intensity activities, whereas children showed they could more accurately identify the two extremes of intensity (ie, sedentary behaviors and vigorous-intensity activities). It could be expected that as a result of children’s sporadic and explosive patterns of activity [3,72-75], moving from one extreme intensity to another, could explain, in part, the present findings demonstrating children’s limited ability to identify the intermediary light- and moderate-intensity activities. Furthermore, the present study showed that only 25% (n=3) of children thought that working on the computer was a physical activity, and none of the children characterized “sweeping” as a sedentary behavior as compared to the previous findings of 38% and 30%, respectively [59]. Although Trost et al [59] encompassed a larger sample of children aged 9-10 years, such discrepancies may be, at least in part, due to the reduced sample size and wider age range in our study. Conversely, it could be argued that the timeframe in which the research was implemented could play an influencing role on youths’ understandings of physical activity. For example, since the mid-2000s, the number of campaigns with mass media components have led to an increased level of exposure to the importance of physical activity behaviors within youths, with evidence supporting this exposure-response relationship [76-78].

The majority of children tended to overestimate light-intensity activities such as stretching exercises, playing darts, and fishing. In some cases, children would associate stretching exercises with other more demanding activities such as warm-up before “...rugby...” (PB07). This type of category contamination was a recurring theme among children, with other activities such as throwing (light) and climbing trees (vigorous) being associated with “...running around” (PB01). In most cases, this category contamination led to an increase in intensity of the dominating activity (ie, going from light to moderate intensity). Furthermore, light-intensity activities such as darts and fishing were often inaccurately identified because of the perceived skill or competence required to complete the activity. Specifically, playing darts was considered a vigorous-intensity activity, as it required a certain skill to “...get [the dart] in the middle [of the dart board]” (PG05), and fishing was associated with moderate intensity because it is “...quite hard to catch fish” (PB02). Skill level was identified as a common characteristic for other activities including football, hockey, swimming, riding a bicycle, and climbing trees, with one child stating reading as a vigorous intensity because “...you have to learn how to read words” (PB06). Consistent with previous findings [27], this study emphasizes that skill in an activity, or physical competence, influenced children’s perceptions of the intensity level. It could be speculated that children’s perspectives of these skill- or physical competence-associated activities are likely to undergo change and refinement as a consequence of time with personal experience and maturation [70]. However, this perspective appears unlikely, as evidence suggests that adults also lack relevant knowledge in terms of determining intensities of physical activity [23]. This demonstrated inability to define intensities further highlights the importance of educating youths about different intensities of physical activity, so that as they age, their understanding of physical activity is more likely to reflect the actual intensity associated with the recommended guidelines.

It is important that youths understand the type of physical activities that form moderate- and vigorous-intensity levels to increase their chances of engaging with these type of activities and gaining the associated metabolic health benefits [79-81]. In the present study, youths demonstrated a limited ability to correctly identify moderate-intensity activities, although the degree of this inaccuracy was much greater in adolescents. It could be postulated that youths’ inability to identify moderate-intensity activities could be aligned with their limited capacity to describe how a physical activity could be performed at different intensities or effort levels [60]. However, youths underestimated the intensity levels of moderate activities related to either household chores, such as sweeping and mowing the lawn, or the daily activity of climbing stairs. Adolescents described such moderate activities as “...everyday things like mowing the lawn” (SG09) and “...like it’s easy” (SB04), and children suggested that when climbing stairs, “...all you’ve got to do is lift a foot and put it on each step” (PB09). These findings support those of Trost et al [59], as household chores and climbing stairs are not considered important contributory sources of physical activity, with the present study further highlighting that this underestimation increased with age. Nonetheless, it is perhaps pertinent to consider the applicability of some activities such as household chores, as a lack of familiarity may have led to exaggerated inaccuracies in the intensity of these activities [82]. For adolescents, the more commonly performed individual sports (swimming, running, and cycling) were correctly identified as vigorous intensity, with team sports such as football, hockey, and tennis perceived to be moderate intensity. Indeed, evidence suggests that the more the activity is considered as play or fun, the less likely youths are aware of the intensity [27]. Although there is limited evidence of this within the present findings, it could be speculated that the greater level of social interaction during team sports [83] and the perceived conception of these team activities for play or fun could function as a moderator to youths’ ability to correctly assess the respective intensity [27].The present findings highlight the need to further understand how context (ie, social settings) mediates youths’ ability to interpret intensities between team sports and the more individual-pursuit sports. Additionally, research is warranted to investigate the potential differences between nonathletic youths’ and sports-orientated youths’ understanding and ability to conceptualize intensities.

Inconsistencies in youths’ ability to correctly identify and understand different activity intensities observed in this study are important, especially given that self-report physical activity questionnaires rely on youths’ ability to correctly interpret activities in accordance with the intensity level [27]. The reliability and validity of data derived from measuring youth’s physical activity using self-report questionnaires is problematic [28-30]. From the present findings, it could be postulated that the inconsistent reliability and validity of physical activity questionnaires are, at least in part, due to youths’ misinterpretations and lack of understanding of intensity, supporting the findings of LeBlanc and Janssen [84]. Indeed, the findings support the idea that youths are not accustomed to relating their physical activity by intensity as a result of limited understanding, which makes it challenging to conduct interventions aimed at changing intensities of physical activity. Additionally, although direct comparisons between sexes failed to demonstrate any significant difference in the ability to align activities with their respective activity intensities, the present findings suggest that girls, irrespective of age, outperformed boys. For example, adolescent girls outperformed their counterparts in correctly identifying light-, moderate-, and vigorous-intensity activities. Interestingly, girls in the younger age group outperformed boys in correctly identifying sedentary and light-intensity activities, although the imbalance in the number of girls (n=3) to boys (n=9) may limit such comparisons. Indeed, these preliminary findings could be explained by differences in cognitive development, as girls have better verbal and written language skills [85-88] and acquire vocabulary faster [89] than boys until adulthood. As a consequence, it could be postulated that the higher level of vocabulary among girls may give them an advantage in aligning activities with intensities. This is especially pertinent because previous research has shown that girls provide more reliable and valid recollections in physical activity questionnaires than boys [90]. More research is warranted to determine whether these verbal and written advantages among girls indeed play a significant role in the understanding of physical intensities, how this may impact self-report questionnaires, and how to best account for these sex differences.

One method that has the potential to develop youths’ comprehension of physical activity levels and associated activity intensities is the use of personalized tangible interfaces (ie, 3D models) to aid learning [91]. The present findings support this notion, with youths demonstrating a good ability to interpret and understand the age-specific 3D models, which is an important step towards enabling a cognitive experience whereby they can start to learn about their physical activity habits [92]. Indeed, previous research has suggested that physical materials can promote playful learning in youth and might offer a more natural interaction than other types of learning interfaces (ie, digital) [46-49]. More specifically, tangible objects can enable collaborative interactions with significant others [93,94], which coincides with an individual achieving social interaction more readily than that from on-screen digital displays [95]. Moreover, evidence suggests that physical activity with the social support of significant others can significantly increase youth’s motivation for physical activity [96] as well as their enjoyment [97,98], intensity [99], and overall engagement in and out of school [100], which holds promise for the 3D models. Equally important, a large proportion of youths (79%) could correctly identify and describe the current physical activity guidelines projected on the 3D models. Youths’ ability to understand the physical activity guideline as a tangible representation will offer a more haptic and proprioceptive experience than visual representations alone [101], which is especially pertinent because youths are regarded as visual and tactile learners [36]. It is anticipated that the 3D models will act as a concept map, whereby youths can make connections and relationships and understand that the concepts about physical activity are not just factual, but rather ideas to increase comprehension and expand vocabulary [102]. Adolescents demonstrated a greater understanding of the age-specific 3D models and the different representations of physical intensities as compared to children, which could be explained by the Piagetian developmental theory [103]. Evidence suggests that the exploratory nature of learning through tangible interfaces such as 3D models of physical activity may offer a more supportive solution to enhancing children’s understanding in identifying patterns (ie, between activities and intensities) and new concepts about physical activity than previous digital methods [91]. In this light, our findings support the use of the age-specific 3D models within a school-based intervention, whereby youths receive a personalized 3D model of their objectively assessed physical activity levels to not only enhance comprehension and understanding of the recommended guidelines and associated intensities, but also use as a unique motivational strategy to increase their physical activity.

### Limitations

Although data saturation was reached and lends further credibility to the findings, this study is limited by the relatively small sample size, age range, and geographical area of data collection, which may underrepresent other socioeconomic groups and ethnic minorities. Furthermore, among children, sex difference comparisons regarding the understanding of intensities may be limited due to the small number of girls who participated in the study. Therefore, our findings on youths’ understanding of the age-specific 3D models and the demonstrated ability to match activities to respective physical intensities should not be generalized but considered a stimulus for future investigation.

### Conclusion

This study shows that both children and adolescents have misconceptions when identifying corresponding activity intensities. Specifically, children showed recurring-intensity classification errors such as category contamination and perceived skill or competence of an activity leading to misperceptions of intensity, with both age groups severely underestimating moderate-intensity activities. However, youths demonstrated a good ability to interpret and describe the age-specific 3D model representations of physical activity, intensity, and the recommended guideline. Therefore, this study highlights the potential utility of these age-specific 3D-printed models within an intervention to act as an educational tool to enhance youths’ understanding and awareness of the recommended physical activity guidelines and associated intensities.

## Abbreviations

MET: metabolic equivalent

## References

[1] Sothern MS, Loftin M, Suskind RM, Udall JN, Blecker U (1999). The health benefits of physical activity in children and adolescents: implications for chronic disease prevention. Eur J Pediatr.

[2] Nieman P (2002). Psychosocial aspects of physical activity. Paediatr Child Health.

[3] Janssen I, Leblanc AG (2010). Systematic review of the health benefits of physical activity and fitness in school-aged children and youth. Int J Behav Nutr Phys Act.

[4] Eime RM, Young JA, Harvey JT, Charity MJ, Payne WR (2013). A systematic review of the psychological and social benefits of participation in sport for children and adolescents: informing development of a conceptual model of health through sport. Int J Behav Nutr Phys Act.

[5] Shiri R, Solovieva S, Husgafvel-Pursiainen K, Telama R, Yang X, Viikari J, Raitakari OT, Viikari-Juntura E (2013). The role of obesity and physical activity in non-specific and radiating low back pain: the Young Finns study. Semin Arthritis Rheum.

[6] Telama R, Yang X, Leskinen E, Kankaanpää A, Hirvensalo M, Tammelin T, Viikari JSA, Raitakari OT (2014). Tracking of physical activity from early childhood through youth into adulthood. Med Sci Sports Exerc.

[7] Wolin K, Yan Y, Colditz G A, Lee IM (2009). Physical activity and colon cancer prevention: a meta-analysis. Br J Cancer.

[8] Department of Health, Physical Activity, Health Improvement and Protection (2011). gov.uk.

[9] Tremblay M, Colley RC, Saunders TJ, Healy GN, Owen N (2010). Physiological and health implications of a sedentary lifestyle. Appl Physiol Nutr Metab.

[10] Biddle S, Cavill N, Ekelund U, Gorely T, Griffiths M, Jago R, Oppert M, Raats M, Salmon J, Stratton G (2010). Sedentary behaviour and obesity: review of the current scientific evidence. The Sedentary Behaviour and Obesity Expert Working Group.

[11] Blair S, LaMonte MJ, Nichaman MZ (2004). The evolution of physical activity recommendations: how much is enough?. Am J Clin Nutr.

[12] (2011). World Health Organization.

[13] Hills A, Andersen LB, Byrne NM (2011). Physical activity and obesity in children. Br J Sports Med.

[14] Townsend N, Wickramasinghe K, Williams J, Bhatnagar P, Rayner M (2015). British Heart Foundation.

[15] Bauman A, Reis RS, Sallis JF, Wells JC, Loos RJF, Martin BW, Lancet Physical Activity Series Working Group (2012). Correlates of physical activity: why are some people physically active and others not?. Lancet.

[16] Carver A, Timperio A, Crawford D (2008). Playing it safe: the influence of neighbourhood safety on children's physical activity. A review. Health Place.

[17] Trost S, Owen N, Bauman AE, Sallis JF, Brown W (2002). Correlates of adults' participation in physical activity: review and update. Med Sci Sports Exerc.

[18] Snethen J, Broome ME (2007). Weight, exercise, and health: children's perceptions. Clin Nurs Res.

[19] Sleap M, Wormald H (2006). Perceptions of Physical Activity Among Young Women aged 16 and 17 Years. European Journal of Physical Education.

[20] Harris J, Cale L, Duncombe R, Musson H (2016). Young people’s knowledge and understanding of health, fitness and physical activity: issues, divides and dilemmas. Sport, Education and Society.

[21] Roth M, Stamatakis E (2010). Linking young people's knowledge of public health guidelines to physical activity levels in England. Pediatr Exerc Sci.

[22] Grewal TK (2013). Electronic Thesis and Dissertation Repository, The University of Western Ontario.

[23] Knox E, Esliger DW, Biddle SJH, Sherar LB (2013). Lack of knowledge of physical activity guidelines: can physical activity promotion campaigns do better?. BMJ Open.

[24] Crossley SGM, McNarry MA, Hudson J, Eslambolchilar P, Knowles ZR, Mackintosh KA (2019). Perceptions of Visualizing Physical Activity as a 3D-Printed Object: Formative Study. J Med Internet Res.

[25] Cowden R, Plowman SA (1999). The Self-Regulation and Perception of Exercise Intensity in Children in a Field Setting. Pediatric Exercise Science.

[26] Prochaska J, Sallis JF, Long B (2001). A physical activity screening measure for use with adolescents in primary care. Arch Pediatr Adolesc Med.

[27] Pearce PF, Harrell JS, McMurray RG (2008). Middle-School Children’s Understanding of Physical Activity: “If You’re Moving, You’re Doing Physical Activity”. Journal of Pediatric Nursing.

[28] Aggio D, Fairclough S, Knowles Z, Graves L (2016). Validity and reliability of a modified english version of the physical activity questionnaire for adolescents. Arch Public Health.

[29] Chinapaw M, Mokkink LB, van Poppel MNM, van Mechelen W, Terwee CB (2010). Physical activity questionnaires for youth: a systematic review of measurement properties. Sports Med.

[30] Martínez-Gómez D, Martínez-de-Haro V, Pozo T, Welk GJ, Villagra A, Calle ME, Marcos A, Veiga OL (2009). [Reliability and validity of the PAQ-A questionnaire to assess physical activity in Spanish adolescents]. Rev Esp Salud Publica.

[31] Brusseau T, Kuilinna PH, Cothran DJ (2011). Health and physical activity content knowledge of Pima children. Physical Educator.

[32] DiClemente C, Marinilli AS, Singh M, Bellino LE (2001). The role of feedback in the process of health behavior change. Am J Health Behav.

[33] Kremers S, Dijkman MA, de Meij JS, Jurg ME, Brug J (2008). Awareness and habit: important factors in physical activity in children. Health Education.

[34] Khot RA, Hjorth L, Mueller FF (2016). Understanding material representations of physical activity. CHI '14 Proceedings of the SIGCHI Conference on Human Factors in Computing Systems.

[35] Ullmer B, Ishii H (2000). Emerging frameworks for tangible user interfaces. IBM Syst. J.

[36] Dunn RS, Dunn KJ (1979). Learning Styles/Teaching Styles: Should They ... Can They ... Be Matched?. Educational Leadership.

[37] Fleming ND, Mills C (1992). Not Another Inventory, Rather a Catalyst for Reflection. To Improve the Academy.

[38] Cole M, Wertsch JV (1996). Beyond the Individual-Social Antinomy in Discussions of Piaget and Vygotsky. Human Development.

[39] Price S, Rogers Y, Scaife M, Stanton D, Neale H (2003). Using ‘tangibles’ to promote novel forms of playful learning. Interacting with Computers.

[40] Marshall P Do tangible interfaces enhance learning?.

[41] Rogers Y, Scaife M, Gabrielli S, Smith H, Harris E A conceptual framework for mixed reality environments: designing novel learning activities for young children.

[42] Bara F, Gentaz E, Colé P, Sprenger-Charolles L (2004). The visuo-haptic and haptic exploration of letters increases the kindergarten-children’s understanding of the alphabetic principle. Cognitive Development.

[43] Khot R, Mueller FF (2013). Sweat-atoms: turning physical exercise into physical objects.

[44] Sauvé K, Houben S, Marquardt S, Bakker S, Hengeveld B, Gallacher S, Rogers Y (2017). LOOP: A physical artifact to facilitate seamless interaction with personal data in everyday life.

[45] Rohit AK, Floyd MF, Larissa H (2013). SweatAtoms: materializing physical activity.

[46] Dourish P (2001). Where the Action Is: The Foundations of Embodied Interaction.

[47] Jacob R J K, Ishii H, Pangaro G, Patten J (2002). A tangible interface for organizing information using a grid.

[48] Triona L, Klahr D (2005). CiteSeerX.

[49] Price S, Rogers Y, Scaife M, Stanton D, Neale H (2003). Using ‘tangibles’ to promote novel forms of playful learning. Interacting with Computers.

[50] Rogers Y, Scaife M, Gabrielli S, Smith H, Harris E (2002). A Conceptual Framework for Mixed Reality Environments: Designing Novel Learning Activities for Young Children. Presence: Teleoperators and Virtual Environments.

[51] Crossley SGM, McNarry M A, Eslambolchilar P, Knowles ZR, Mackintosh KA (2019). The Tangibility of Personalised 3D Printed Feedback May Enhance Youths Physical Activity Awareness, Goal-Setting and Motivation. J Med Internet Res (forthcoming).

[52] Gittelsohn J, Steckler A, Johnson CC, Pratt C, Grieser M, Pickrel J, Stone EJ, Conway T, Coombs D, Staten LK (2006). Formative research in school and community-based health programs and studies:. Health Educ Behav.

[53] Meade C, Calvo A, Cuthbertson D (2002). Impact of culturally, linguistically, and literacy relevant cancer information among Hispanic farmworker women. J Cancer Educ.

[54] Bopp M, Fallon E (2008). Community-based interventions to promote increased physical activity: a primer. Appl Health Econ Health Policy.

[55] Martinez J, Latimer AE, Rivers SE, Salovey P (2012). Formative research for a community-based message-framing intervention. Am J Health Behav.

[56] Druin A (2002). The role of children in the design of new technology. Behaviour & Information Technology.

[57] Kennedy C, Kools S, Krueger R (2001). Methodological considerations in children's focus groups. Nurs Res.

[58] Heary C, Hennessy E (2006). Focus Groups Versus Individual Interviews with Children: A Comparison of Data. The Irish Journal of Psychology.

[59] Trost SG, Morgan AM, Saunders RP, Felton GA, Ward DS (2000). Children 's Understanding of the Concept of Physical Activity. Pediatric Exercise Science.

[60] Ridley K, Ainsworth BE, Olds TS (2008). Development of a compendium of energy expenditures for youth. Int J Behav Nutr Phys Act.

[61] Butte N, Watson KB, Ridley K, Zakeri IF, McMurray RG, Pfeiffer KA, Crouter SE, Herrmann SD, Bassett DR, Long A, Berhane Z, Trost SG, Ainsworth BE, Berrigan D, Fulton JE (2018). A Youth Compendium of Physical Activities: Activity Codes and Metabolic Intensities. Med Sci Sports Exerc.

[62] Evenson K, Catellier DJ, Gill K, Ondrak KS, McMurray RG (2008). Calibration of two objective measures of physical activity for children. J Sports Sci.

[63] Hamad E, Savundranayagam MY, Holmes JD, Kinsella EA, Johnson AM (2016). Toward a Mixed-Methods Research Approach to Content Analysis in The Digital Age: The Combined Content-Analysis Model and its Applications to Health Care Twitter Feeds. J Med Internet Res.

[64] Holsti OR (1969). Content analysis for the social sciences and humanities.

[65] Pool IS (1959). Trends in Content Analysis: A Review Article. Journal of Folklore Research.

[66] Braun V, Clarke V (2006). Using thematic analysis in psychology. Qualitative Research in Psychology.

[67] Boddy L, Knowles ZR, Davies IG, Warburton GL, Mackintosh KA, Houghton L, Fairclough SJ (2012). Using formative research to develop the healthy eating component of the CHANGE! school-based curriculum intervention. BMC Public Health.

[68] Campbell I (2007). Chi-squared and Fisher-Irwin tests of two-by-two tables with small sample recommendations. Stat Med.

[69] Richardson J (2011). The analysis of 2 × 2 contingency tables--yet again. Stat Med.

[70] Brustad R (1991). Children's perspectives on exercise and physical activity: measurement issues and concerns. J Sch Health.

[71] Cardinal B, Engels H, Zhu W (1998). Application of the Transtheoretical Model of Behavior Change to Preadolescents’ Physical Activity and Exercise Behavior. Pediatric Exercise Science.

[72] Sleap M, Warburton P (1996). Physical activity levels of 5-11-year-old children in England: cumulative evidence from three direct observation studies. Int J Sports Med.

[73] Welk G, Corbin CB, Dale D (2000). Measurement issues in the assessment of physical activity in children. Res Q Exerc Sport.

[74] Baquet G, Stratton G, Van Praagh E, Berthoin S (2007). Improving physical activity assessment in prepubertal children with high-frequency accelerometry monitoring: a methodological issue. Prev Med.

[75] Adamo K, Prince SA, Tricco AC, Connor-Gorber S, Tremblay M (2009). A comparison of indirect versus direct measures for assessing physical activity in the pediatric population: a systematic review. Int J Pediatr Obes.

[76] Economos C, Hyatt RR, Goldberg JP, Must A, Naumova EN, Collins JJ, Nelson ME (2007). A community intervention reduces BMI z-score in children: Shape Up Somerville first year results. Obesity (Silver Spring).

[77] Huhman M, Potter LD, Duke JC, Judkins DR, Heitzler CD, Wong FL (2007). Evaluation of a national physical activity intervention for children: VERB campaign, 2002-2004. Am J Prev Med.

[78] Sanigorski A, Bell AC, Kremer PJ, Cuttler R, Swinburn BA (2008). Reducing unhealthy weight gain in children through community capacity-building: results of a quasi-experimental intervention program, Be Active Eat Well. Int J Obes (Lond).

[79] Andersen L, Harro M, Sardinha LB, Froberg K, Ekelund U, Brage S, Anderssen SA (2006). Physical activity and clustered cardiovascular risk in children: a cross-sectional study (The European Youth Heart Study). Lancet.

[80] Ekelund U, Anderssen SA, Froberg K, Sardinha LB, Andersen LB, Brage S, European Youth Heart Study Group (2007). Independent associations of physical activity and cardiorespiratory fitness with metabolic risk factors in children: the European youth heart study. Diabetologia.

[81] Rizzo N, Ruiz JR, Hurtig-Wennlöf A, Ortega FB, Sjöström M (2007). Relationship of physical activity, fitness, and fatness with clustered metabolic risk in children and adolescents: the European youth heart study. J Pediatr.

[82] Li s (2016). The Conversation.

[83] Brettschneider W (2001). Effects of sport club activities on adolescent development in Germany. European Journal of Sport Science.

[84] LeBlanc A, Janssen I (2010). Difference between self-reported and accelerometer measured moderate-to-vigorous physical activity in youth. Pediatr Exerc Sci.

[85] Lynn R (1992). Sex Differences on the Differential Aptitude Test in British and American Adolescents. Educational Psychology.

[86] Mann V, Sasanuma S, Sakuma N, Masaki S (1990). Sex differences in cognitive abilities: a cross-cultural perspective. Neuropsychologia.

[87] Martin D, Hoover HD (2016). Sex Differences in Educational Achievement: A Longitudinal Study. The Journal of Early Adolescence.

[88] Undheim J, Nordvik H (1992). Socio‐economic Factors and Sex Differences in an Egalitarian Educational System: academic achievement in 16‐year‐old Norwegian students. Scandinavian Journal of Educational Research.

[89] Roulstone S, Loader S, Northstone K, Beveridge M (2002). The Speech and Language of Children Aged 25 Months: Descriptive Data from the Avon Longitudinal Study of Parents and Children. Early Child Development and Care.

[90] Rangul V, Holmen TL, Kurtze N, Cuypers K, Midthjell K (2008). Reliability and validity of two frequently used self-administered physical activity questionnaires in adolescents. BMC Med Res Methodol.

[91] Marshall P (2007). Do tangible interfaces enhance learning?.

[92] Forlizzi J, Battarbee K (2004). Understanding experience in interactive systems.

[93] Fernaeus Y, Tholander J (2006). “Looking At the Computer but Doing It On Land”: Children’s Interactions in a Tangible Programming Space.

[94] Suzuki H, Kato H (1995). Interaction-level support for collaborative learning: AlgoBlock—an open programming language.

[95] Svendsen G (1991). The influence of interface style on problem solving. International Journal of Man-Machine Studies.

[96] Salvy S, Roemmich JN, Bowker JC, Romero ND, Stadler PJ, Epstein LH (2009). Effect of peers and friends on youth physical activity and motivation to be physically active. J Pediatr Psychol.

[97] Jago R, Page AS, Cooper AR (2012). Friends and physical activity during the transition from primary to secondary school. Med Sci Sports Exerc.

[98] Salvy S, de la Haye Kayla, Bowker Julie C, Hermans Roel C J (2012). Influence of peers and friends on children's and adolescents' eating and activity behaviors. Physiol Behav.

[99] Barkley J, Salvy SJ, Sanders GJ, Dey S, Von Carlowitz KP, Williamson ML (2014). Peer influence and physical activity behavior in young children: an experimental study. J Phys Act Health.

[100] Pearce M, Page AS, Griffin TP, Cooper AR (2014). Who children spend time with after school: associations with objectively recorded indoor and outdoor physical activity. Int J Behav Nutr Phys Act.

[101] Gillet A, Sanner M, Stoffler D, Olson A (2005). Tangible interfaces for structural molecular biology. Structure.

[102] Butzow C, Butzow JW (1990). Science through Children's Literature: An Integrated Approach. Science Activities: Classroom Projects and Curriculum Ideas.

[103] Piaget J, Cook M (1952). The origins of intelligence in children.

